# 1例复合杂合突变导致遗传性凝血因子Ⅺ缺陷症的分子致病机制

**DOI:** 10.3760/cma.j.cn121090-20230814-00065

**Published:** 2024-03

**Authors:** 元 陈, 朗译 秦, 双女 林, 丽红 杨, 柯 张, 龙颖 叶, 艳慧 金, 明山 王

**Affiliations:** 温州医科大学附属第一医院医学检验中心，浙江省检验诊断及转化研究重点实验室，温州 325015 Department of Clinical Laboratory, Key Laboratory of Clinical Laboratory Diagnosis and Translational Research of Zhejiang Province, the First Affiliated Hospital of Wenzhou Medical University, Wenzhou 325015, China

## Abstract

1例34岁女性患者因“乳房结节”拟行手术切除，术前检查发现活化部分凝血活酶时间（APTT）66.2 s、凝血因子Ⅺ活性（FⅪ∶C）2％、FⅪ抗原（FⅪ∶Ag）40.3％，患者及家系成员均无异常出血表现。诊断为遗传性凝血因子Ⅺ缺陷症。基因检测发现其F11基因第10外显子c.1107C>A（p.Tyr351stop）杂合无义突变、第13外显子c.1562A>G（p.Tyr503Cys）杂合错义突变，其父亲和儿子为p.Tyr351stop突变的杂合携带者，而母亲和女儿为p.Tyr503Cys突变的杂合携带者。体外表达结果显示，p.Tyr351stop突变导致F11基因转录水平显著降低，而p.Tyr503Cys突变对F11基因转录水平以及蛋白表达水平无影响，但该突变导致细胞培养上清液中FⅪ∶C水平显著降低。

## Abstract (en)

A 34 year old female patient was scheduled to undergo surgical resection due to a "breast nodule". Preoperative examination revealed an activated partial thromboplastin time（APTT）of 66.2 seconds, coagulation factor Ⅺ activity（FⅪ: C）of 2%, and FⅪ antigen（FⅪ: Ag）of 40.3%. The patient and family members showed no abnormal bleeding symptoms. Diagnosed as hereditary coagulation factor Ⅺ deficiency. Genetic testing revealed that the F11 gene had a heterozygous nonsense mutation in exon 10, c.1107C>A（p.Tyr351stop）, and a heterozygous missense mutation in exon 13, c.1562A>G（p.Tyr503Cys）. The father and son were p Heterozygous carriers of Tyr351stop mutation, while the mother and daughter are p Heterozygous carriers of Tyr503Cys mutations. The in vitro expression results showed that p The Tyr351stop mutation resulted in a significant decrease in the transcription level of F11 gene, while p The Tyr503Cys mutation has no effect on the transcription level and protein expression level of F11 gene, but it leads to a significant decrease in the level of FⅪ:C in the cell culture supernatant.

凝血因子Ⅺ（FⅪ）是由肝脏细胞合成，以2条结构相同的单体通过二硫键连接形成的同源二聚体形式存在于血液中[1]–[2]。每个单体均由一条含有四个重复的苹果结构域（AP1～4）的重链以及一个含有催化活性中心的轻链构成。不同AP结构域的功能各不相同，其中AP4结构域中的Cys321位点是两个单体通过二硫键结合形成同源二聚体的部位。同源二聚体结构是FⅪ蛋白分泌至细胞外的先决条件，位于该结构域的突变可能会影响FⅪ蛋白二聚体的形成，从而导致FⅪ单体在细胞内滞留[3]–[4]。分泌进入血液中的成熟FⅪ蛋白主要由凝血酶或活化的凝血因子Ⅻ（FⅫa）通过裂解p.Arg369-p.Ile370之间的肽键被激活为FⅪa，进而参与止血功能[5]。本研究通过对一个导致FⅪ缺陷症的复合杂合突变突变体进行体外表达研究，探讨其可能的分子致病机制，并为该类疾病的研究提供理论和实验依据。

## 对象与方法

1. 家系资料：先证者，女，34岁，浙江省瑞安市人，2021年2月因“乳房结节”于温州医科大学附属第一医院就诊，术前检查发现活化部分凝血活酶时间（APTT）66.2 s（参考值29.0 s～40.3 s）、FⅪ活性（FⅪ∶C）2％（参考值82％～122％）、FⅪ抗原（FⅪ∶Ag）40.3％（参考值76％～127％），其他凝血指标均无明显异常，肝肾功能正常。先证者妊娠两胎并自然分娩，产后24 h总失血量均在450 ml左右，无月经量过多或异常出血病史。其余家系成员平素均无异常出血表现。

选择2023年4月于温州医科大学附属第一医院体检中心100名健康体检者作为健康对照组，其中男性58名，女性42名，年龄23～52岁，均无肝肾功能异常、出血及血栓形成病史。本研究通过温州医科大学附属第一医院伦理委员会审查（伦审KY2022-R193），共涉及先证者家系6人及100名健康对照者，所有受试者于实验前均签署知情同意书。

2. 标本采集及样本处理：所有受试者外周静脉全血均采集于0.109 mol/L枸橼酸钠抗凝管中，1∶9抗凝。3 000 r/min离心10 min后，分离上层乏血小板血浆用于凝血指标的检测，下层血细胞用于基因组DNA的提取。

3. 凝血功能检查：上层乏血小板血浆在Stago-STA-R-Max全自动凝血仪（法国Diagnostica Stago公司及配套试剂）上采用一期凝固法检测血浆凝血酶原时间（PT）、APTT、FⅧ∶C、FⅨ∶C、FⅪ∶C和FⅫ∶C。使用温州长风生物技术有限公司的酶联免疫吸附试验（ELISA）试剂盒检测血浆FⅪ∶Ag含量。

4. 外周血基因组DNA提取及测序：采用血液基因组DNA提取试剂盒（购自北京天根生化科技有限公司）提取先证者及其家系成员外周血基因组DNA。于ABI Thermal cycler 2720扩增仪（美国Thermo Fisher Scientific公司产品）上扩增F11基因共15个外显子及其侧翼序列，引物设计及扩增条件参考文献[6]。扩增后的PCR产物送上海赛恒生物科技有限公司纯化后直接测序。测序结果采用Chromas软件与NCBI数据库中公布的人类F11基因（ID:2160）参考序列进行对比，寻找突变位点。证实先证者突变位点后，对其家系成员相应突变位点区域进行测序分析，明确其是否存在相应的突变位点。

5. 生物信息学分析：采用Mutation Taster（https://www.mutationtaster.org/），PolyPhen-2（http://genetics.bwh.harvard.edu/pph2/index.shtml），PROVEAN和SIFT（https://www.jcvi.org/research/provean），LRT（http://varcards.biols.ac.cn/），CADD（http://cadd.gs.washington.edu）在线生物信息学分析软件对突变位点的致病性进行预测。

6. 构建野生型和突变型FⅪ表达载体：以pCDH-cop GFP-T2A-Puro质粒为模板构建FⅪ蛋白表达载体，空载质粒和野生型FⅪ蛋白表达载体（FⅪ-WT）购买自杭州擎科生物技术有限公司。使用Primer X软件（http://www.bioinformatics.org/primerx/）设计突变引物，根据QuikChange Lightning Site-Directed Mutagenesis Kit定点突变试剂盒（美国STRATAGENE公司）说明书在FⅪ-WT的基础上构建FⅪ-p.Tyr351stop和FⅪ-p.Tyr503Cys突变型表达载体。构建的突变型FⅪ蛋白表达载体送杭州擎科生物技术有限公司测序确定。

7. 细胞培养和转染：解冻复苏HEK293T细胞，使用含有10％胎牛血清的DMEM完全培养基于37 °C含5％ CO_2_的环境中培养。使用瞬时转染试剂LipofectamineTM 3 000 reagent（美国Thermo Fisher Scientific公司产品）将空载质粒、FⅪ-WT、FⅪ-p.Tyr351stop以及FⅪ-p.Tyr503Cys表达载体转染生长状态良好的HEK293T细胞，并于48 h后于荧光显微镜下观察转染效率。

8. F11基因转录水平的检测：瞬时转染48 h后，使用RNAiso Plus试剂（日本TaKaRa公司）提取转染细胞内总RNA，采用HiScriptⅡQ RT SuperMix for qPCR试剂（中国南京诺唯赞生物科技股份有限公司）将总RNA逆转录为cDNA。根据Taq Pro Universal SYBR qPCR Master Mix试剂（中国南京诺唯赞生物科技股份有限公司）说明书，通过实时荧光定量PCR法（qRT-PCR）检测转染细胞中野生型和突变型F11基因mRNA表达水平。采用Bio-Rad CFX Manager 3.1软件分析F11基因和GAPDH基因的Ct值，利用比较Ct法（2^−ΔΔCt^法）计算野生型和突变型F11基因mRNA的相对表达量。F11基因和GAPDH基因引物序列（表1）。

**表1:** F11和GAPDH基因引物序列

基因名称	正向序列	反向序列
*F11*	5′-GCAACGAAGGGAAGGGCAA-3′	5′-CCTGGGCTTGATTTTGGTGG-3′
*GAPDH*	5′-AGAAGGCTGGGGCTCATTTG-3′	5′-AGGGGCCATCCACAGTCTTC-3′

9. FⅪ蛋白表达水平的检测：收集转染阳性细胞，用含PMSF的RIPA缓冲液裂解细胞。细胞培养上清液和细胞裂解液中的蛋白变性后通过10％ SDS聚丙烯酰胺凝胶电泳、转膜、封闭以及一抗二抗孵育后显影检测。采用ELISA法检测细胞培养上清液和细胞裂解液中的FⅪ∶Ag水平，ELISA试剂盒购买自上海江莱生物科技有限公司。采用一期凝固法检测细胞培养上清液中的FⅪ∶C水平，并以FⅪ∶C/FⅪ∶Ag的值为特异活性。

## 结果

1. 凝血功能检查结果：先证者APTT延长至66.2 s，FⅪ∶C和FⅪ∶Ag分别降低至2％和40.3％，其父亲、母亲、儿子和女儿的APTT均略有延长，其中先证者父亲和儿子的FⅪ∶C和FⅪ∶Ag同步降低，其母亲和女儿的FⅪ∶C降低而FⅪ∶Ag在参考范围内（表2）。

**表2:** 先证者及其家系成员实验室表型及基因型检测结果

家系成员	PT（s）	APTT（s）	FⅧ∶C（%）	FⅨ∶C（%）	FⅪ∶C（%）	FⅪ∶Ag（%）	FⅫ∶C（%）
先证者	13.9	66.2	151	101	2	40.3	63
Ⅰ_1_	12.5	45.9	218	114	54	43.5	87
Ⅰ_2_	12.1	47.3	178	98	59	95.7	79
Ⅱ_2_	12.8	31.2	119	118	103	109	90
Ⅲ_1_	13.5	45.2	121	87	48	97.2	82
Ⅲ_2_	13.2	43.1	131	96	52	46.1	90
参考值	11.5~14.8	29.0~43.0	80~134	72~136	84~122	76～127	72~114

注 PT：凝血酶原时间；APTT：活化部分凝血活酶时间；FⅪ∶Ag：凝血因子Ⅺ抗原；FⅧ∶C、FⅨ∶C、FⅪ∶C、FⅫ∶C分别为凝血因子Ⅷ、Ⅸ、Ⅺ、Ⅻ活性

2. 基因测序结果：基因检测结果表明，先证者F11基因第10外显子存在c.1107C>A杂合无义突变（rs773905328），导致p.Tyr351stop，第13号外显子存在c.1562A>G杂合错义突变（HGMD号：CM185272），导致p.Tyr503Cys；其父亲和儿子均为p.Tyr351stop突变的杂合携带者，而母亲和女儿均为p.Tyr503Cys突变的杂合携带者，其丈夫为F11基因野生型。家系图见图1，测序图见图2。

**图1:**
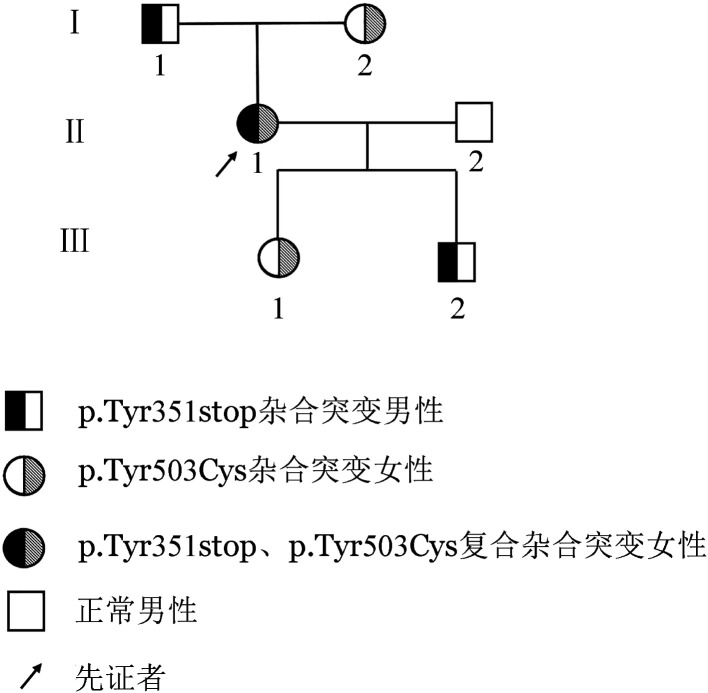
遗传性FⅪ缺陷症家系图

**图2:**
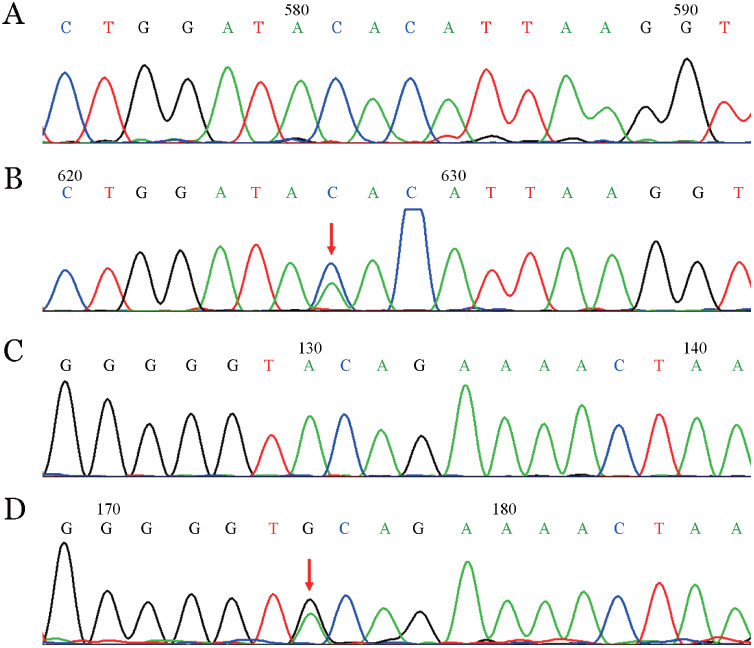
F11基因第10号和第13号外显子测序结果 A：c.1107C>A野生型；B：正向测序图，箭头示c.1107C>A杂合无义突变; C：c.1562A>G野生型；D：正向测序图，箭头示c.1562A>G杂合错义突变

3. 生物信息学预测结果：Mutation Taster、LRT以及CADD在线生物信息学分析软件对p.Tyr351stop突变的预测结果分别为“致病的”、“有害的””以及“损害的”，得分为1、0以及34分。六个在线生物信息学分析软件对p.Tyr503Cys突变的预测为“致病的”、“可能致病的”、“损害的”、“损害的”、“有害的”以及“损害的”，得分为0.998、1.000、−3.350、0.035、0以及29.200。此外，p.Tyr351stop突变在人群中的等位基因频率为3.251e-05。

4. 体外表达实验结果：qRT-PCR结果表明，转染HEK293T细胞48 h后与野生型相比，FⅪ-p.Tyr351stop突变型F11 基因mRNA的表达水平显著降低，而FⅪ-p.Tyr503Cys突变型与野生型F11基因mRNA表达水平差异无统计学意义（*P*＝0.7836），提示p.Tyr503Cys突变不影响F11基因的转录（图3）。ELISA法检测显示，以FⅪ-WT表达载体转染的HEK293T细胞的细胞培养上清液和裂解液中的FⅪ∶Ag含量为100％，同样以该样本培养上清液中FⅪ∶C和FⅪ∶C/FⅪ∶Ag比值为100％。检测发现，与FⅪ-WT相比，FⅪ-p.Tyr351stop表达载体细胞培养裂解液和培养上清液中FⅪ∶Ag含量分别为（16.0±13.6）％和（5.2±1.8）％，而FⅪ-p.Tyr·503Cys表达载体的细胞裂解液和培养上清液中FⅪ∶Ag含量分别为（109.2±9.1）％和（92.3±2.8）％，但其FⅪ∶C/FⅪ∶Ag仅为FⅪ-WT的（10.7±4.3）％。Western blot结果显示，与FⅪ-WT相比FⅪ-p.Tyr351stop表达载体细胞裂解液和培养上清液中均检测不到FⅪ蛋白的存在，而FⅪ-p.Tyr503Cys表达载体细胞裂解液和培养上清液中FⅪ蛋白的含量与野生型（WT）无显著差异（图4）。

**图3:**
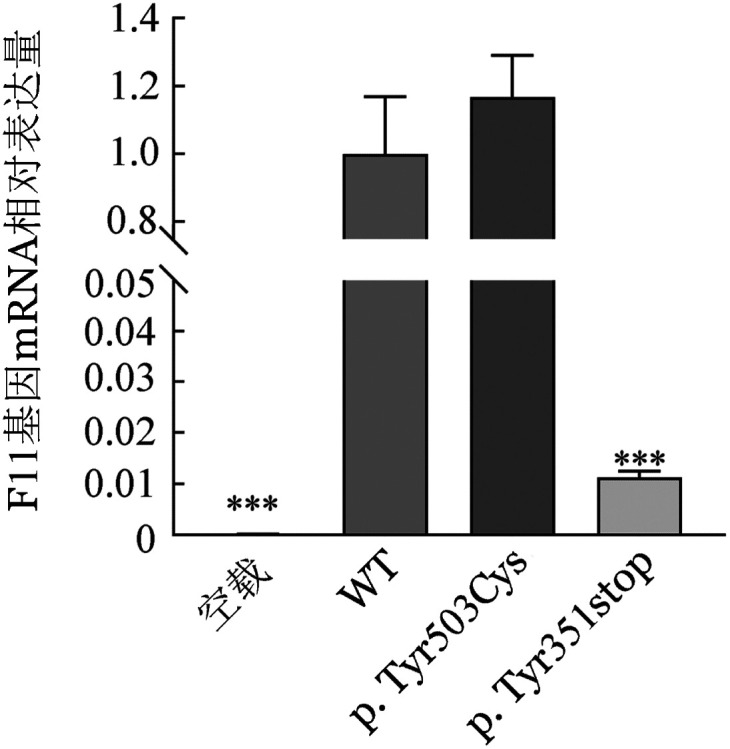
转染细胞内mRNA表达的qRT-PCR检测结果（***P<0.001）

**图4:**
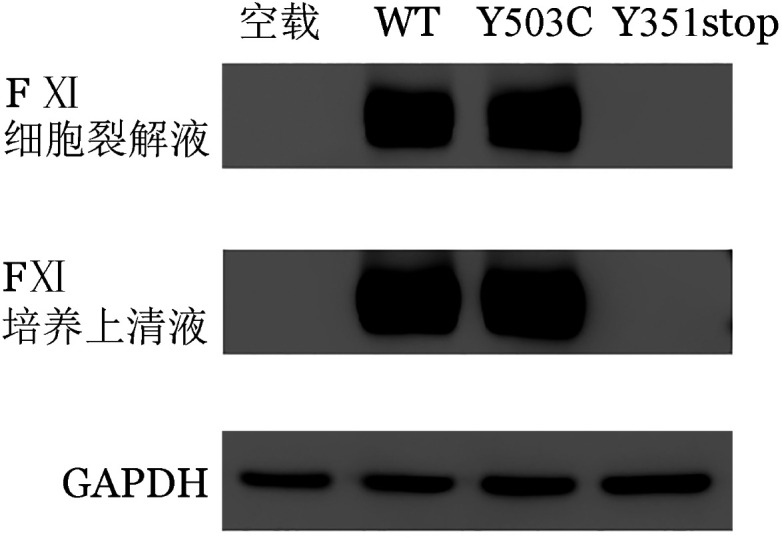
Western blot法检测细胞裂解液和培养上清液中凝血因子Ⅺ蛋白表达

## 讨论

遗传性FⅪ缺陷症是由位于常染色体4q35上的F11基因突变导致的不完全隐性遗传性疾病，其年发病率为1/100万～1/10万[7]。根据血液中FⅪ∶C和FⅪ∶Ag降低程度的差异可将其分为两型：Ⅰ型为交叉反应物质阴性（CRM^−^），表现为FⅪ∶C和FⅪ∶Ag同步降低；Ⅱ型为交叉反应物质阳性（CRM^+^），表现为FⅪ∶C降低而FⅪ∶Ag正常[8]。遗传性FⅪ缺陷症患者多无自发性出血症状或症状轻微，但在高纤溶活性的组织或器官（如甲状腺、扁桃体、前列腺）损伤或手术时可有出现难止的倾向，而其他低纤溶活性部位手术或创伤则很少出现过量出血[9]。这可能是由于血小板内贮存的FⅪ分泌进入血液中并且在止血过程中发挥重要作用的原因[10]。此外，近年来有研究发现FⅪ也与血栓形成相关，血浆中高水平的FⅪ是深静脉血栓形成的高危因素之一，而严重的FⅪ缺陷患者的静脉血栓发生率较低，证明严重的FⅪ缺陷对血栓疾病起保护作用[11]–[12]。因此有临床研究将预防和治疗血栓的活化FⅪ（FⅪa）抑制剂用于血栓栓塞患者的抗凝治疗中，通过比较发现与传统新型口服抗凝药相比FⅪa抑制剂对于血栓患者症状的缓解可能更加有效并且可以减少出血并发症的发生[13]。

本研究中，先证者FⅪ∶C和FⅪAg分别降低至2％和40.3％，经基因检测发现其F11基因第10号外显子及第13号外显子分别存在p.Tyr351stop杂合无义突变以及p.Tyr503Cys杂合错义突变。而先证者父亲和儿子均为p.Tyr351stop杂合子携带者，他们的FⅪ∶C和FⅪ∶Ag同步降低，属于Ⅰ型FⅪ缺陷症；其母亲和女儿均为p.Tyr503Cys杂合子携带者，他们的FⅪ∶C降低而FⅪ∶Ag正常，属于Ⅱ型FⅪ缺陷症。结合先证者表型检测结果表明，其同时具有Ⅰ型和Ⅱ型FⅪ缺陷症特征。家系调查研究证明参与本研究的家族成员均无肝肾功能异常及其他可能导致FⅪ缺陷症的疾病存在。因此，我们初步认为p.Tyr351stop杂合无义突变以及p.Tyr503Cys杂合错义突变是该家系FⅪ水平降低的主要原因。

p.Tyr351stop杂合突变最初由Au等[14]在一个拔牙后持续出血的中国女性家系调查研究中发现，他们认为该突变可能是其FⅪ∶C降低的原因，但并没有对其具体致病机制进行研究。本研究通过构建FⅪ-p.Tyr351stop表达载体转染HEK293T细胞发现，与转染WT的细胞相比，转染该载体的细胞内mRNA转录显著减少，同时细胞裂解液和培养上清液中均检测不到FⅪ蛋白以及FⅪ∶Ag的存在。该结果显示p.Tyr351stop突变提前产生的终止密码子可能触发了无义介导的mRNA降解途径（NMD）。NMD途径是广泛存在于真核细胞内的高度保守的mRNA降解机制，主要用于检查可能产生无功能和/或显性负蛋白的常见的错误mRNA，并将其从转录组中切割和消除，从而保持基因表达的完整性[15]。因此，本研究认为p.Tyr351stop突变产生的异常mRNA可能会被NMD途径识别及降解，进而导致FⅪ蛋白合成的减少。

轻链上的催化活性中心由空间结构催化三联体（His413-Asp462-Ser557）构成，该结构对于FⅪ蛋白的催化活性具有至关重要的作用，研究表明发生在此处的突变可能直接影响FⅪ∶C[16]。p.Tyr503位于FⅪ蛋白的催化三联体结构域中，目前已有报道发现p.Tyr503残基的移码突变（p.Tyr503Valfs*32）破坏催化结构域，导致催化结构域破坏[17]。p.Tyr503Cys突变最早本课题组报道[18]，初步研究认为该突变可能是通过二硫键错配导致FⅪ蛋白催化结构域的破坏引起FⅪ∶C活性降低。但为进一步确定p.Tyr503Cys突变的具体致病机制，本研究构建了FⅪ-p.Tyr503Cys表达载体瞬时转染HEK293T细胞，结果表明p.Tyr503Cys突变不会影响F11基因的转录以及FⅪ蛋白的合成和分泌，但与转染FⅪ-WT表达载体的细胞相比其上清液中FⅪ蛋白的特异活性明显降低。因此，认为p.Tyr503Cys突变可能通过影响催化三联体结构域导致FⅪ蛋白催化活性降低。

综上所述，本研究报道了1例复合杂合突变p.Tyr351stop和p.Tyr503Cys导致的遗传性FⅪ缺陷症及其家系。通过体外表达实验证明，p.Tyr351stop突变导致F11基因mRNA加速降解；p.Tyr503Cys突变导致FⅪ蛋白催化活性降低。对这两个突变的深入研究有助于加强对FⅪ缺陷症机制的深入了解，为FⅪa抑制剂的研究提供新思路。
